# Protective efficacy of recombinant canine adenovirus type-2 expressing TgROP18 (CAV-2-ROP18) against acute and chronic *Toxoplasma gondii* infection in mice

**DOI:** 10.1186/s12879-015-0815-1

**Published:** 2015-03-04

**Authors:** Xiu-Zhen Li, Xiao-Hu Wang, Li-Jun Xia, Ya-Biao Weng, Jorge A Hernandez, Li-Qing Tu, Lu-Tao Li, Shou-Jun Li, Zi-Guo Yuan

**Affiliations:** College of Veterinary Medicine, South China Agricultural University, Guangzhou, Guangdong Province 510642 PR China; Guangdong Provincial Key Laboratory of Prevention and Control for Severe Clinical Animal Diseases, Guangzhou, Guangdong Province 510642 PR China; Institute of Animal Health, Guangdong Academy of Agricultural Sciences, Guangzhou, Guangdong Province 510642 PR China; Department of Large Animal Clinical Sciences, College of Veterinary Medicine, University of Florida, Gainesville, Florida USA

**Keywords:** *Toxoplasma gondi*, Recombinant virus CAV-2-ROP18, Protective immunity, Mice

## Abstract

**Background:**

The use of recombinant viral vectors expressing *T. gondii* antigens is a safe and efficient approach to induce immune responses against the parasite, as well as a valuable tool for vaccine development. We have previously prolonged the survival time of mice challenged with the RH strain of *T. gondii* by immunizing the mice with a eukaryotic vector expressing the protein ROP18 of *T. gondii*. We are now looking for ways to improve this vaccination strategy and enhance protection.

**Methods:**

In this study, we constructed and characterized a novel recombinant canine adenovirus type 2 expressing ROP18 (CAV-2-ROP18) of *T. gondii* by cytopathic effect (CPE) and indirect immunofluorescence assay (IFA) following transfection into MDCK cells. Intramuscular immunization of Kunming mice with CAV-2-ROP18 was carried out to evaluate humoral and cellular immune responses.

**Results:**

The vaccination of experimental mice with CAV-2-ROP18 elicited antibody production against ROP18, including high levels of a mixed IgG1/IgG2a and significant production of IFN-γ or IL-2, and displayed a significant bias towards a helper T cell type 1 (Th1) profile. Furthermore, the presence of *T. gondii*-specific IFN-γ-production and TNF-α-production T cells was elicited in both CD^4+^ and CD^8+^ T cell compartments. Significantly higher survival rates (40%) occurred in the experimental group, and a reduction in brain cyst burden was detected in vaccinated mice.

**Conclusion:**

These results demonstrate the potential use of a CAV vector harboring the ROP18 gene in the development of a vaccine against acute and chronic toxoplasmosis.

**Electronic supplementary material:**

The online version of this article (doi:10.1186/s12879-015-0815-1) contains supplementary material, which is available to authorized users.

## Background

*Toxoplasma gondii* is an obligate, intracellular parasite which belongs to the phylum Apicomplexa [1]. The parasite can infect all warm-blooded mammals. In humans it is one of the major opportunistic parasites that infects immunocompromised individuals and pregnant women [2-4], causing congenital defects in newborns and severe, disseminated disease in adults. Toxoplasmosis also causes considerable economic losses in livestock, especially in pigs and sheep [5]. Chemical treatments for acute and chronic toxoplasmosis are currently available, but they are not acceptable due to parasite drug-resistant and chemical residues in food [6,7]. Because of the public health and eco2nomic consequences of *T. gondii* infection in humans and animals, the development of a vaccine is needed for disease prevention.

The *T. gondii* ROP18 protein is a polymorphic serine-threonine kinase which is secreted in the host cell during the invasion process, and its catalytic activity is required for the acute virulence phenotype. ROP18 is considered one of the key virulence factors in the pathogenesis of the T. gondii infection [8,9]. Previous research has demonstrated that an additional ligand-binding pocket outside of the active site cleft is a key element of the ROP18 Ser/Thr protein kinase for mediating acute virulence in mice [10].

The use of recombinant viral vectors has great potential for the development of more immunogenic vaccines against protozoan parasites. Viral vectors typically elicit efficient expression of the foreign antigens they encode, which facilitate the presentation and development of specific immune responses against the recombinant antigen [11,12]. Here we describe the development of a recombinant canine adenovirus expressing the ROP18 gene of *T. gondii* that partially protected mice against challenge with the RH strain (genotype I) and Prugniaud (PRU) strain (genotype II) of *T. gondii.*

## Methods

### Mice, cell and parasites

One hundred and thirty-two specific-pathogen-free (SPF) grade, female, inbred Kunming mice, 6 to 8 weeks old, were purchased from the Sun Yat-Sen University Laboratory Animal Center. All mice were handled properly according to the Animal Ethics Procedures and Guidelines of the People’s Republic of China. The study was approved by the Animal Ethics Committee of South China Agricultural University (permit SCAUAEM-2013-39). All mice were maintained under standard conventional conditions, with food and water *ad libitum*.

Madin-Darby canine kidney cell lines (MDCK) were cultivated and two *T. gondii* strains (RH and PRU) were used in our lab (see Additional file 1).

### The construction of pPolyII-CAV-ΔE3-ROP18

**Figure 1:**
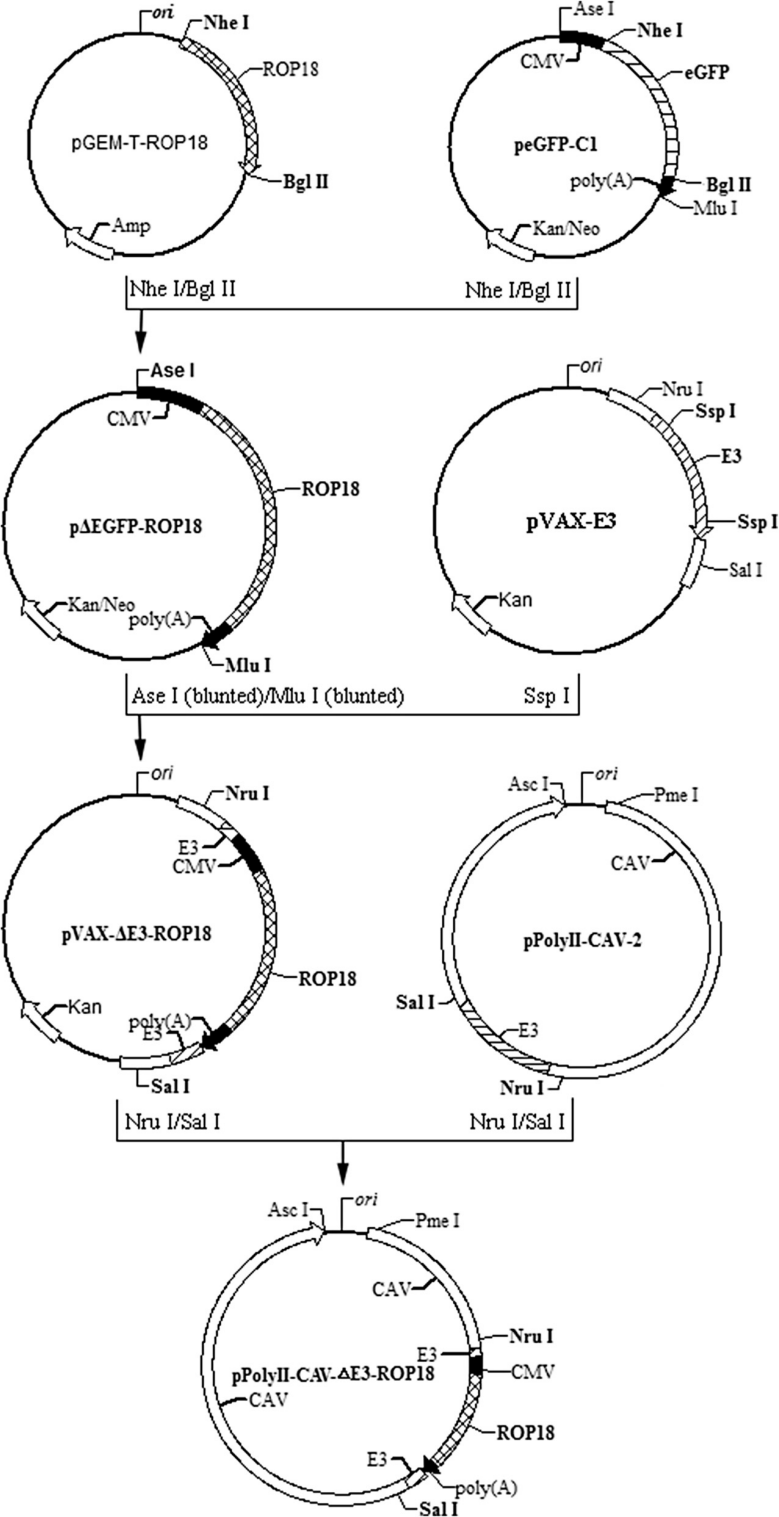
**Schematic representation of the construction of recombinant plasmid pPolyII-CAV-△E3-ROP18 by in vitro ligation.** E3, the E3 region of CAV-2; CMV, human cytomegalovirus (hCMV) immediate-early gene promoter; polyA, the SV40 early mRNA polyadenylation signal. Bold letters were those enzymes used in plasmid construction.

The construction of pPolyII-CAV-ΔE3-ROP18 (Figure 1) was performed as described in Additional file 2.

### Transfection of recombinant genome in MDCK cells and identification of ROP18 expression from CAV-2-ROP18

Five micrograms of pPolyII-CAV-ΔE3-ROP18 were digested with Asc I and Pme I to release the linear recombinant genome. After extraction with chloroform and precipitation with ethanol, the recombinant genome was used to transfect MDCK cells at 70–80% confluency with Lipofectamine 2000^TM^ (Invitrogen). The transfected MDCK cells were passaged routinely until a typical CAV-2 cytopathic effect (CPE) was observed.

For identification of the expression of ROP18 by recombinant CAV-2-ROP18, the indirect immunofluorescence assay (IFA) was done as reported in Additional file 3 [4].

### Vaccination procedure and challenge

All mice were randomly assigned into one of four experimental groups (33 mice per group). Group I was intramuscularly inoculated once with 0.1 ml CAV-2-ROP18 (10 ^8.125^ p.f.u. ml^−1^); group II received 0.1 ml CAV-2 (10^8.25^ p.f.u. ml^−1^) intramuscularly once as a negative control; group III was inoculated intramuscularly with 0.1 ml PBS as control at weeks 0, 2 and 4; and group IV was not injected with anything as a negative control. Blood was collected from the lateral saphenous vein of a hind limb of 5 mice per group one day prior to each immunization and at intervals of two weeks after inoculation.Sera were separated and stored at -20°C until analyzed for specific antibodies. Pre-immune sera were used as negative controls.

Eight weeks after the immunization, 20 mice in each group were challenged intraperitoneally (i.p.) with 1 × 10^3^ tachyzoites of the virulent *T. gondii* RH strain, and 10 other mice were inoculated intragastrically with 5 cysts of the PRU strain. All mice were observed daily for mortality. Two months after the challenge, the surviving mice were euthanized and their brains were removed. Each brain was homogenized in 2 ml of PBS. The mean number of cysts per brain was determined by counting in three samples of 25 μl aliquots of each homogenized brain under an optical microscope.

### Humoral response

Levels of antigen-specific IgG, IgG1 and IgG2a immunoglobulins in serum samples were examined as previously described (see Additional file 4) [13].

CAV-2 hemagglutination inhibition (HI) antibody titers were determined by a micro method with a slight modification (see Additional file 5) [14].

### Lymphocyte proliferation assay

At week 8 (post-immunization), splenocytes were harvested from each of the three immunized mice from each group, separately. Next, the spleen cells proliferative response was measured as mentioned above (for details, see Additional file 6) [4,15,16].

### Cytokine assays

Detection of cytokines was carried out according to the method previously described (see Additional file 7 for details) [4].

### Evaluation of CTL activity

Peripheral blood mononuclear cells (PBMCs) of mice were segregated from 3 mice per group and single-cell suspensions were prepared from mice 8-weeks after the immunization, and the activity of cytotoxic T lymphocytes (CTL) was measured by CytoTox 96® Non-Radioactive Cytotoxicity Assay Kits (Promega, USA) as previously reported (see Additional file 8) [15].

### Flow cytometry analysis

Co-expression of CD3+ with CD4^+^ and CD8^+^ on lymphocytes of splenocytes were determined by flow cytometry as previously mentioned (see Additional file 9) [13].

Detection of intracellular cytokines was carried out according to the method of Additional file 9.

### Statistical analysis

All data were processed and analyzed by SPSS13.0 Data Editor (SPSS Inc., Chicago, IL, USA). Mean antibody responses, lymphoproliferation, cytokine production and CTL were compared between groups by using one-way ANOVA. If data are not normally distributed, the non-parametric Kruskal-Wallis test was used in the study. Values of *P < 0.05* were considered significant.

## Results

### Recovery and identification of CAV-2-ROP18

**Figure 2:**
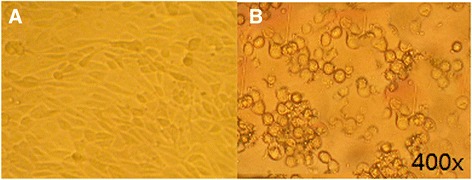
**Cytopathic effects on MDCK cells after transfection (×400).** Normal cells **(A)**; transfected and cytopathic cells **(B)**.

**Figure 3:**
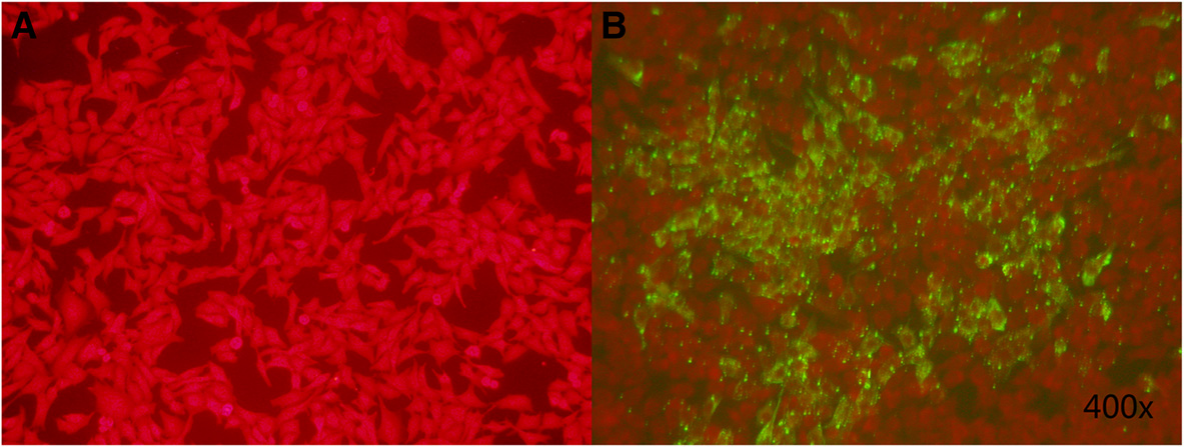
**Expression analyses of ROP18 protein in transfected MDCK cells by IFA at 48 h post-transfection with a presumptive recombinant virus CAV-2-ROP18: (A) Non-transfected MDCK cells; (B) MDCK cells detected with** ***T. gondii*** **-positive serum post-transfection with a presumptive recombinant virus CAV-2-ROP18.** The pictures were showed under 400× microscope.

Seven days after transfection of the recombinant genome pPolyII-CAV-ΔE3-ROP18 into MDCK cells, typical adenovirus-like CPE (grape-cluster-like cells) was observed under an optical microscope (200×, Figure 2). The growth characteristics of the recombinant virus was similar to that of the canine adenovirus vaccine strain YCA18 (data not shown). The identification of the recombinant virus genome by restriction endonuclease digestion and PCR amplification confirmed that the ROP18 gene and its expression cassette were included in the recombinant virus (data not shown). IFA demonstrates expression of ROP18 following transduction of MDCK cells with CAV2-ROP18, whereas no fluorescence was observed in negative control MDCK cells by use of an anti-ROP18 polyclonal antiserum (Figure 3).

### Evaluation of humoral responses

**Figure 4:**
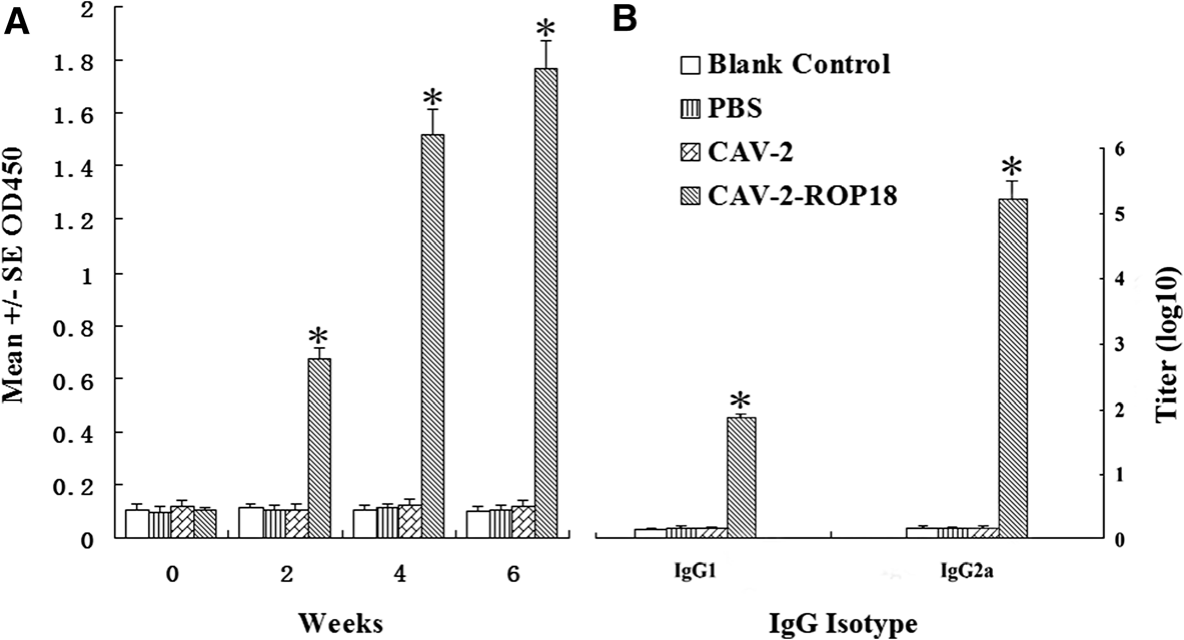
**Detection of CAV-2-ROP18 immunization on the antibody response.** Serum samples were collected at weeks 0, 2, 4 and 6 post-primary immunization. **(A)** Determination of specific anti-ROP18 IgG antibodies in the sera of Kunming mice. **(B)** Determination of the specific anti-ROP18 IgG subclass profile in the sera of the immunized Kunming mice. Results are expressed as means of the OD_490_ value and standard deviation (n = 5), and statistically significant difference (*P* < 0.05) are indicated by an asterisk (*).

Using ELISA, antibody titers significantly increased in the recombinant virus CVA-2-ROP18 group at week 2, 4 and 6 after immunization, compared to CAV-2, PBS and blank control immunized group (*P <* 0.05) (Figure 4A). Subclasses of immunoglobulins specific to *T. gondii* were analyzed in the sera (as shown in Figure 4B). Both IgG1 and IgG2a were tested in sera of mice immunized with CAV-2-ROP18, and the ratios of IgG1 to IgG2a in the groups I - IV were 2.69, 1.02, 1.08, and 1.09, respectively. There was no significant difference in IgG1 and IgG2a levels between the groups immunized CAV-2, PBS, and nothing (*P* > 0.05). It is known that production of IgG subclasses is driven by cytokines secreted during cellular immune responses. Therefore, the presence of IgG1 and IgG2a indirectly suggests that the vaccination protocol also promoted activation of a cell-mediated response.

HI antibodies against CAV-2 were detected in all mice vaccinated with CAV-2 and the recombinant CAV-2-ROP18 at 2 weeks post-primary immunization, reaching comparable titers throughout the test period. In addition, HI titers were up to 1:128 eight weeks after primary vaccination (Table 1). In mice vaccinated with PBS and those not vaccinated, specific antibodies to CAV-2 were not detected.

**Table 1:** **CAV-2 HI antibody titers in mice immunized with PBS, CAV-2 and CAV-2-ROP18**

**Group (n = 3)**	**0 week**	**2 weeks**	**4 weeks**	**6 weeks**	**8 weeks**	**10 weeks**
CAV-2-ROP18	<1:2	1:8-1:16	1:16-1:32	1:64	1:128	1:64-1:128
CAV-2	<1:2	1:8-1:16	1:16-1:32	1:64	1:128	1:64-1:128
PBS	<1:2	<1:2	<1:2	<1:2	<1:2	<1:2
Blank Control	<1:2	<1:2	<1:2	<1:2	<1:2	<1:2

### Cellular immune response analysis

Splenocytes from mice immunized with CAV-2-ROP18 showed a very significant proliferative response to ROP18 (*P* < 0.05), and splenocyte proliferation was ∼ 21-fold higher than proliferation by splenocytes from groups immunized with CAV-2, PBS and negative control (*P* < 0.05). Meanwhile, splenocytes from all groups proliferated to comparable levels in response to the mitogen ConA (Table 2). Significant CTL activity was tested in mice immunized with CAV-2-ROP18 (at E:T value of 100:1; Figure 5).

**Table 2:** **Cytokine production by splenocytes of immunized mice after stimulation by ROP18 protein at 8-weeks post immunization** ^*^

**Group (n = 3)**	**Cytokine production (pg/mL)**				**Proliferation (Stimulation Index)** ^**#**^	
	**IFN-γ**	**IL-2**	**IL-4**	**IL-10**	**ROP18**	**ConA**
CAV-2-ROP18 (I)	914.26 ± 36.56^a^	431.07 ± 28.94^a^	197.29 ± 29.98^a^	44.37 ± 38.54^a^	4.87 ± 0.65^a^	2.93 ± 0.43^a^
CAV-2 (II)	44.28 ± 9.37^b^	47.23 ± 7.59^b^	52.65 ± 9.24^b^	38.49 ± 5.48^a^	0.23 ± 0.06^b^	3.07 ± 0.19^a^
PBS (III)	46.13 ± 10.27^b^	52.60 ± 11.97^b^	54.87 ± 9.45^b^	41.36 ± 5.23^a^	0.26 ± 0.10^b^	2.81 ± 0.32^a^
Blank Control (IV)	42.98 ± 6.76^b^	51.45 ± 3.88^b^	53.38 ± 5.97^b^	40.63 ± 6.74^a^	0.27 ± 0.07^b^	2.84 ± 0.26^a^

*Data are reported as mean ± SD.

^#^Splenocytes from mice were collected 8-weeks after the immunization.

Values for IL-10 and IFN-γ are for 72 h, values for IL-2 and IL-4 are for 24 h.

Within each column, groups with different superscripts are different (*P* < 0.05).

**Figure 5:**
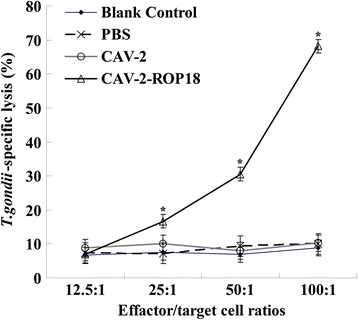
**CTL activity of splenocytes from vaccinated mice 8 weeks after the first immunization.** These splenocytes were tested for ROP18-specific CTL activity of ROP18 target cells loaded with CAV-2-ROP18 (triangle), CAV-2 (circle), PBS (crossing) or nothing (diamond). Data shown represent one experiment of three performed with similar results. E/T ratios are indicated on the horizontal axis. The vertical axis shows *T.gondii*-specific lysis as a percentage of the total possible lysis (% specific lysis).

**Figure 6:**
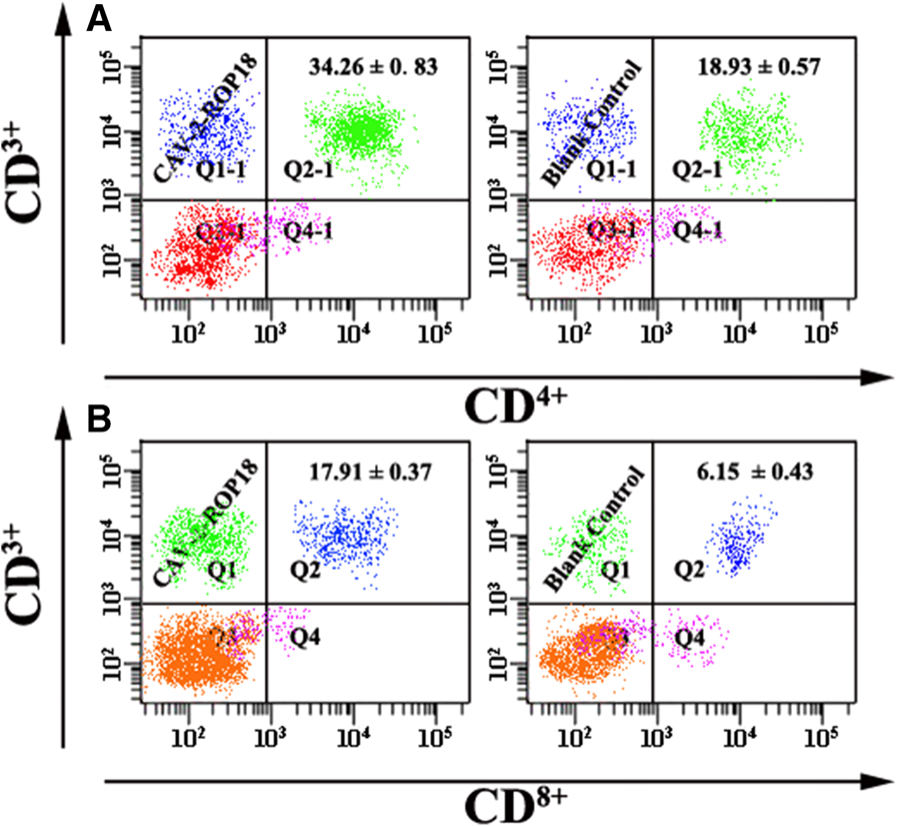
**Analysis of lymphocyte subpopulations (CD** ^**3+**^ **/CD** ^**4+**^ **of CD** ^**3+**^ **/CD** ^**8+**^ **) responses in Kunming mice vaccinated with CAV-2-ROP18 or nothing. (A)** The percentages of CD^3+^/CD^4+^ T lymphocytes. **(B)** The percentages of CD^3+^/CD^8+^ T lymphocytes. Data are reported as mean ± SD.

**Figure 7:**
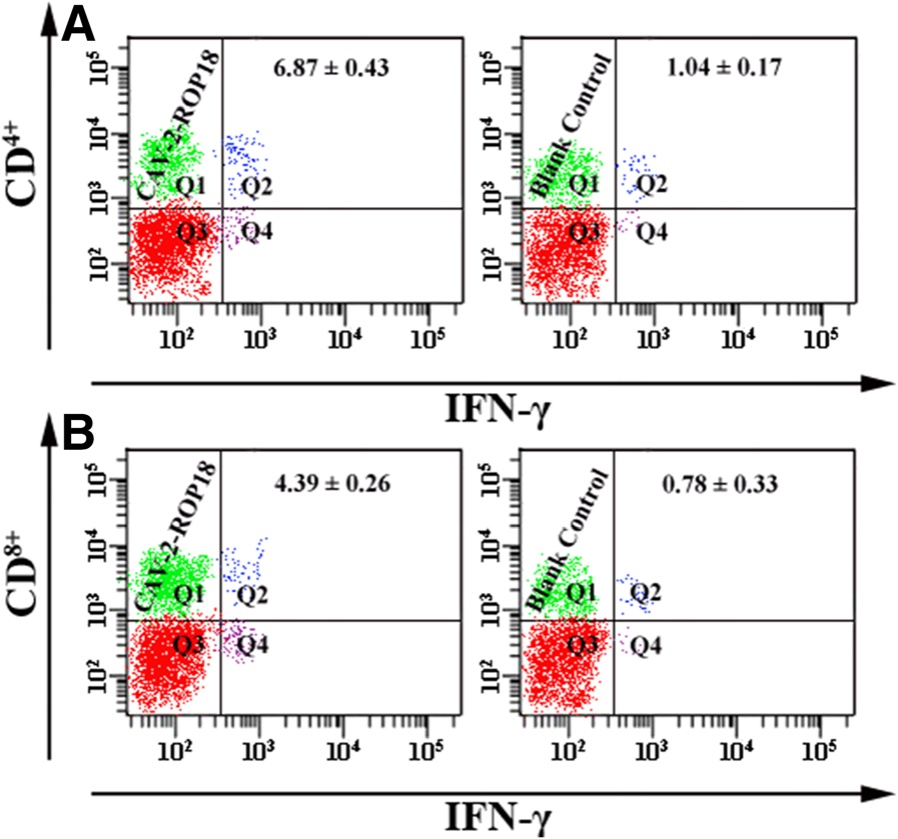
**The expression of IFN-γ on CD** ^**4+**^ **and CD** ^**8+**^ **T cells using flow cytometry analysis.** The percentages of IFN-γ-producting cells inside CD^4+^ T cell gate **(A)** and IFN-γ-producting cells inside CD^8+^ T cell gate **(B)** in mice spleen cells. Data are reported as mean ± SD.

**Figure 8:**
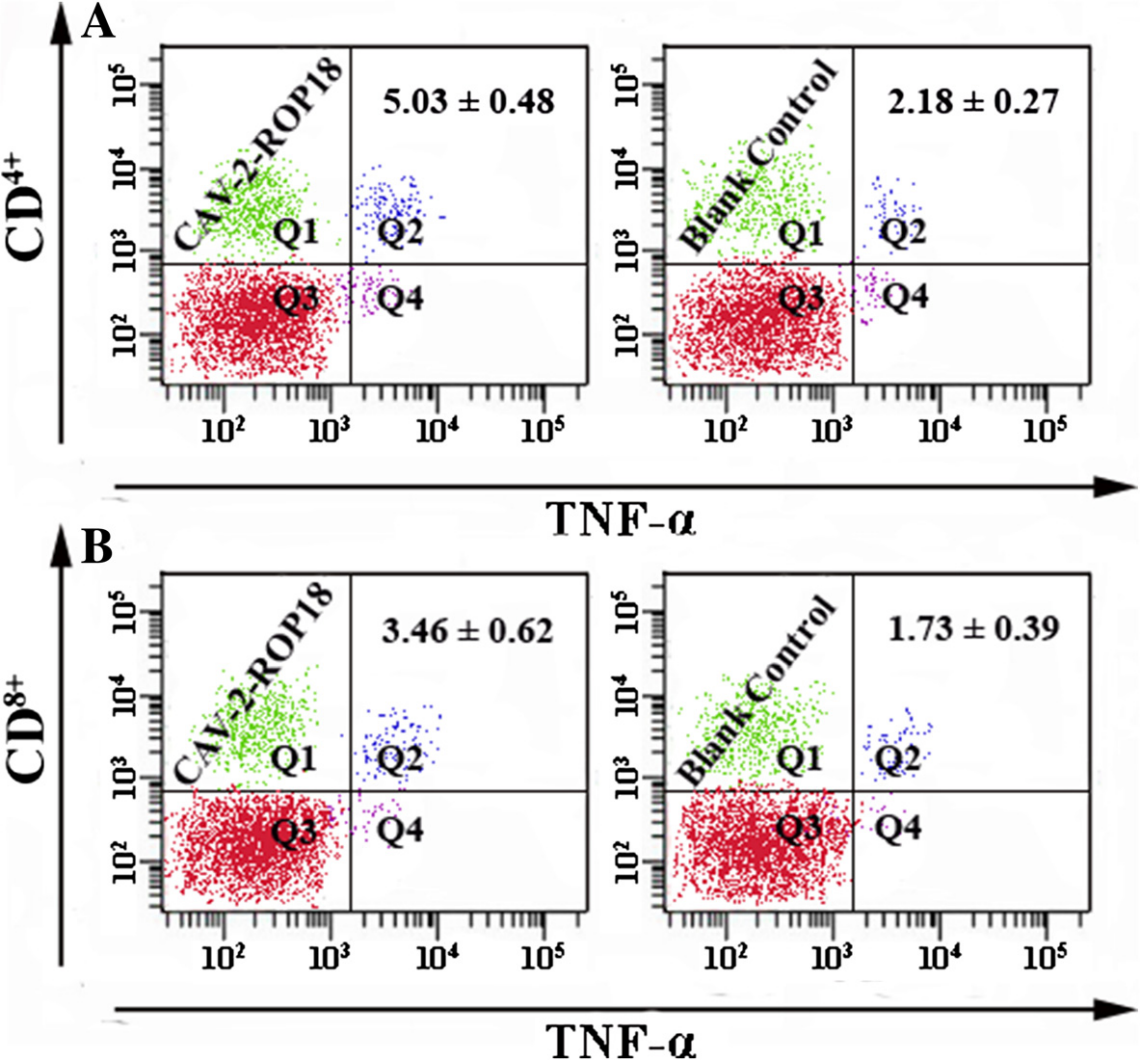
**The expression of TNF-α on CD** ^**4+**^ **and CD** ^**8+**^ **T cells using flow cytometry analysis. A**, percentages of TNF-α-producting cells inside CD^4+^ T cell gate. **B**, percentages of TNF-α-producting cells inside CD^8+^ T cell gate. Data are reported as mean ± SD.

For further characterization of cellular immune responses, the CD3^+^/CD4^+^ T cells and CD3^+^/CD8^+^ T cells were shown in Figure 6.The percentage of CD3^+^/CD4^+^ T cells and CD3^+^/CD8^+^ T cells were significantly increased in mice immunized with CAV-2-ROP18 compared to those with CAV-2, PBS, or blank control groups. Similarly, recombinant virus immunization CAV-2-ROP18 significantly altered CD4^+^ or CD8^+^ T cell profiles in terms of IFN-γ (Figure 7) and TNF-α (Figure 8) expression in comparison with all controls. There was no significant difference between the three control groups (*P* > 0.05).

### Cytokine production

The cell-mediated immunity induced in the immunized mice was further evaluated by measuring the amount of cytokines IL-2, IL-4, IL-10 and IFN-γ. As shown in Table 2, values of IL-2 and IFN-γ in CAV-2-ROP18 immunization group are 431.07 ± 28.94 pg/ml and 914.26 ± 36.56 pg/ml, which are very significantly higher than in the control groups (CAV 47.23 ± 7.59 pg/ml and 44.28 ± 9.37 pg/ml; PBS 52.60 ± 11.97 pg/ml and 46.13 ± 10.27 pg/ml; blank control 51.45 ± 3.88 pg/ml and 42.98 ± 6.76 pg/ml) (*P* < 0.05). For IL-4, low levels of IL-4 showed a slight but significantly production from the splenocytes from mice immunized with CAV-2-ROP18, compared to three control groups (*P* < 0.05), further confirming the results of the IgG subclass. On the other hand, no statistically significant differences could be found in the amount of IL-10 between the immunized and control groups (*P* > 0.05).

### Protection of mice against challenge with *T. gondii*

**Figure 9:**
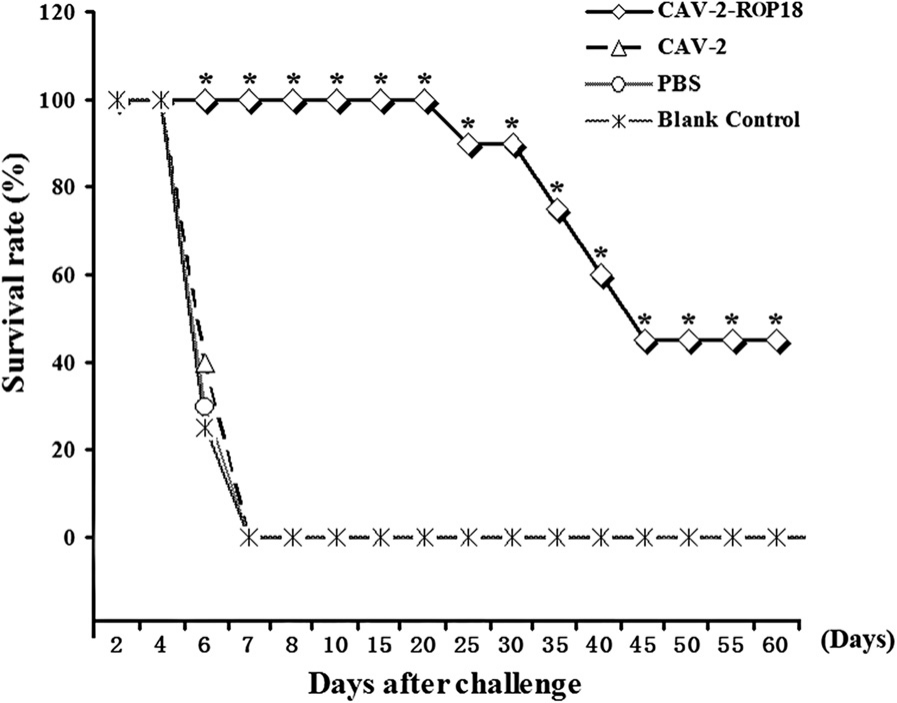
Survival rate of mice immunized with CAV-2-ROP18 (◊), CAV-2 (Δ), PBS (☼) and nothing (*), then challenged with 1 × 10 ^**3**^ **tachyzoites.** Each group had 20 mice. Statistically significant differences (*P* < 0.05) are indicated by an asterisk (*), which of CAV-2-ROP18 are listed differently compared to the other other control groups.

To examine protective immunity, 20 mice of each group were given an intraperitoneal injection of 1 × 10^3^ tachyzoites of *T. gondii* RH strain at 8 weeks after vaccination. Mortality was checked daily. The percentages of survival in the different groups of mice are shown in Figure 9. Those mice immunized with only a single dose of CAV-2-ROP18 showed 40% protection until 60 days after challenge. The administration of either CAV-2 or PBS did not prevent mortality (mice died within 7 days). Also, we evaluated the immunoprotective effect by counting the number of cysts in a chronic model challenge with 5 cysts of strain PRU administration by gavage. As shown in Table 3, we observed that mice from the CAV-2-ROP18 vaccination group developed a significantly lower (*P* < 0.05) number of brain cysts (8000 ± 1414 cysts per brain) in comparison to mice from the other three control control groups (approximately 18000 cysts).

**Table 3:** **Mean cyst burden per mouse brain 30 days after injection with5 cysts of PRU strain by intragastrical route**

**Group (n = 5)**	**No. of brain cysts** ^*^	**Reduction (%)** ^**#**^
CAV-2-ROP18 (I)	8000 ± 1414^a^	57.3
CAV-2 (II)	17480 ± 1584^b^	6.6
PBS (III)	18080 ± 642^b^	3.4
Blank Control (IV)	18720 ± 955^b^	-

*Data are reported as mean ± SD.

^a,b^The same letter indicates no difference (P > 0.05), whereas different letters indicate a significant difference (P < 0.05).

^#^From the values for the blank control.

## Discussion

In this study, a recombinant CAV-2 genome carrying the ROP18 expression cassette was first constructed, and the recombinant adenovirus was then generated by transfecting MDCK cells with the recombinant genome. Regarding the vaccine vectors used for ROP18 expression of *T. gondii*, we started our work with the replication-competent CAV. Besides being highly efficient for transgene expression “*in vivo*” and being safe for administration, adenoviral vectors also have intrinsic adjuvant properties capable of activating an innate immune response via TLR [17,18] and NLR [19] receptors. Meanwhile, several different species of adenovirus have been developed by homologous recombination with insertion of foreign genes into the non-essential E3 region [20,21]. Replication-defective adenoviruses have often been utilized as candidate vaccine vectors. However, clinical application of the best-studied human adenovirus type-5 (AdHu5) is limited by the high prevalence of preexisting immunity resulting from natural infection [22,23]. Many researchers are apprehensive about the problem of a long-acting vaccine in the animal body, because the antibodies could be produced when the animals were infected by CAV in nature. In this study we used the CAV-2 as a vector to express the ROP18 antigen, because CAV-2 is an artificial domestic strain [24], and previous exposure to CAV-2 is low [25]. In the study, titers of HI antibodies to CAV-2 induced by the recombinant virus CAV-2-ROP18 were similar to those induced by CAV-2 vaccine strain, which indicates that the recombinant virus retains its ability to stimulate an effective immune response against CAV.

We also evaluated the Th1 and/or Th2 immune response in mice immunized by prime-boost strategy. The IFN-γ secreted by Th1 cells favors the IgG2a switch, while IL-4 produced by Th2 cells regulates the IgG1 switch [26]. The development of potent Th1-type immune response is essential for the control of *T. gondii* infection [27,28]. As the indicator for activated Th1 lymphocytes, the pro-inflammatory cytokines including IFN-γ and IL-2 are also involved in the protection against the infection [29,30]. Our results showed that in contrast with the controls, immunization with CAV-2-ROP18 induced the production of high levels of IL-2 and IFN-γ (as well as IgG2a), which are associated with Th1-type mediated immunity. Nevertheless, Th2-type cytokines, both IL-4 and IL-10, may partially inhibit the secretion of pro-inflammatory cytokines and prevent CD4^+^ T cell-mediated severe immunopathology during the acute and chronic stage of *T. gondii* invasion [31]. The production of IL-4 in early infection inhibits protective Th1 cell differentiation most likely by either direct or indirect inhibition of IFN-γ production [32]. A slight increase in the release of IL-4 (as well as IgG1) in combination with the high levels of IL-2 and IFN-γ suggested the activation of an appropriate T helper response, primarily a specific Th1-biased cellular immune response after immunization with CAV-2-ROP18. There was no difference in IL-10 production between vaccinated and control groups. The results suggested that Th1-mediated cellular immunity in the group immunized with CAV-2-ROP18 is not dependent on IL-10 production during the cellular response against toxoplasmosis.

In addition to cellular immunity, humoral immunity resulting in the production of antigen-specific IgG antibodies also seems to be important in controlling *T. gondii* invasion [33]. It has been reported that *T. gondii* infection could lead to B cell responses resulting in production of antibodies, which limit the spread of parasites by inhibiting the attachment of tachyzoites to host cell receptors and thus promoting intracellular killing of antibody-coated parasites by macrophages [33]. Our study found that the mice immunized with CAV-2-ROP18 significantly increased the antigen-specific IgG compared with the mice immunized with CAV-2 or PBS. This result indicated that adenovirus vector is highly efficient for gene transfer and expression.

Previous studies demonstrated that DNA vaccine expressing *T. gondii* antigens could elicit both humoral and cell-mediated immunity and prolong the survival time in mice [4,13,15,34-37]. However, no protection was observed in mice, regardless of DNA vaccine or recombinant virus vaccine [4,13,15,34-37]. In our study, we improved the survival rate, which is up to 40% protection in mice immunized with CAV-2-ROP18 after challenge with lethal RH strain of *T. gondii*. PRU strain (genotype II) of *T. gondii* was used in our research in order to scientifically evaluate immunoprotection while using many more mice in this test. We also obtained a 57.3% reduction in brain cysts after immunization of mice with CAV-2-ROP18. Furthermore, the cross-protective immunity has been demonstrated, despite the existing 7.5% sequence variation between type I (RH strain) and type II (PRU strain).

CAV-2-ROP18 could elicit antigen-specific CD4^+^ T cells and CD8^+^ T cells responses in mice. However, their major advantage at the immunological level has been their capacity to induce antigen-specific CD8^+^ T cell responses, including CTLs, which is a major mechanism of protection against intracellular pathogens. Some studies have shown that CTLs are involved in cyst control during *T. gondii* infection in a mechanism mediated by perforin [38]. Meanwhile, it is very important that CD8^+^ T cells, particularly in synergy with CD4^+^ T cells, contributed to the control of the spreading and development of *T. gondii* infection [39,40]. In agreement with this efficacy, we observed the increase of the percentage of T CD8^+^ and T CD4^+^ cells in mice immunized with CAV-2-ROP18, which also suggested the activation of CD4^+^ and CD8^+^ T cells, and thus may be in synergy to contribute to cytotoxic activity against *T. gondii*. Additionally, the present study showed that CD4^+^ and CD8^+^ T cells were up-regulated after the administration of CAV-2-ROP18, at least in terms of frequency of both type cells producing IFN-γ of TNF-α, which was observed in cytotoxicity assay *in vitro*. The CTL activity in the group immunized with CAV-2-ROP18 was significantly higher than that of the group injected with pVAX-ROP18 alone, as previously described (*T. gondii*-specific lysis is 68% vs 47%) [13].

## Conclusions

In summary, our work presents the successful use of recombinant virus CAV-2-ROP18 in vaccination protocols to protect against intraperitoneal and intragastrical challenge with virulent RH strain or attenuated PRU strain of *T. gondii*. This system was shown to be extremely efficient in eliciting humoral and cellular immune responses. Therefore, the CAV-2-ROP18 may be potentially useful in the development of an effective vaccine against *T. gondii* infection in the future.

## Additional files

Additional file 1:
**Cell and parasites.**
Additional file 2:
**The construction of pPolyII-CAV-ΔE3-ROP18.**
Additional file 3:
**The indirect immunofluorescence assay.**
Additional file 4:
**Humoral response.**
Additional file 5:
**Hemagglutination inhibition.**
Additional file 6:
**Lymphocyte proliferation assay.**
Additional file 7:
**Cytokine assays.**
Additional file 8:
**CTL activity.**
Additional file 9:
**Flow cytometry detection.**
