# Supplementary material for: Protective efficacy of recombinant canine adenovirus type-2 expressing TgROP18 (CAV-2-ROP18) against acute and chronic Toxoplasma gondii infection in mice
Source: BMC Infect Dis. 2015 Mar 4;15:114. doi: 10.1186/s12879-015-0815-1 (PMC4397727; doi:10.1186/s12879-015-0815-1)
Supplement: Additional file 1: — Cell and parasites. [file 12879_2015_815_MOESM1_ESM.doc]

**Supplementary Material 1**

[Madin-Darby canine kidney](http://www.google.com.hk/url?sa=t&rct=j&q=&esrc=s&source=web&cd=8&cad=rja&ved=0CEkQFjAH&url=http%3A%2F%2Fwww.ncbi.nlm.nih.gov%2Fpubmed%2F9874698&ei=JNnBUuLqO6SI2wWHlYCgAw&usg=AFQjCNEysgOMzlUgD2cO3iKVD1ZGCjbi5w) cells (MDCK) purchased from Chinese Institute of Veterinary Drug Control, Beijing, China. The MDCK cell lines were grown and maintained in Dulbecco’s modified Eagle’s medium (Invitrogen) supplemented with 10 % (v/v) heat-inactivated fetal calf serum (Gibco Life Technologies), 100 mg streptomycin ml^-1^ and 100 IU penicillin ml^-1^ at 37℃5% CO_2_.

Tachyzoites of the highly virulent RH strain of *T.gondii* were preserved in our laboratory (Laboratory of Parasitology, College of Veterinary Medicine, South China Agricultural University) and maintained by serial intraperitoneal passage in Kunming mice. Brain tissue cysts of strain PRU were obtained from the brains of Kunming mice 1 month after intraperitoneal infection with 5 cysts.
