# Supplementary material for: Protective efficacy of recombinant canine adenovirus type-2 expressing TgROP18 (CAV-2-ROP18) against acute and chronic Toxoplasma gondii infection in mice
Source: BMC Infect Dis. 2015 Mar 4;15:114. doi: 10.1186/s12879-015-0815-1 (PMC4397727; doi:10.1186/s12879-015-0815-1)
Supplement: Additional file 2: — The construction of pPolyII-CAV-ΔE3-ROP18. [file 12879_2015_815_MOESM2_ESM.doc]

**Supplementary Material 2**

**
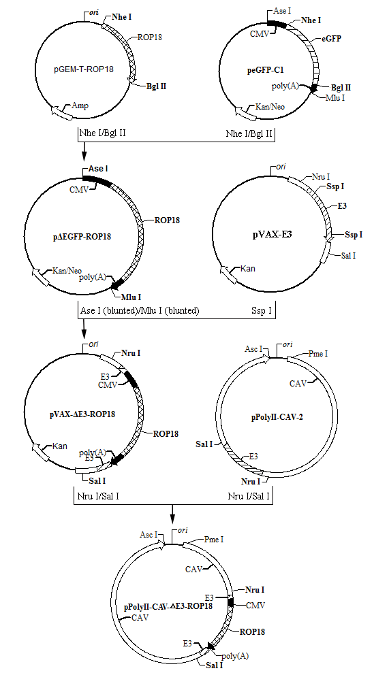
**

**Fig. 1.** Schematic representation of the construction of recombinant plasmid pPolyII-CAV-△E3-ROP18 by in vitro ligation. E3, the E3 region of CAV-2; CMV, human cytomegalovirus (hCMV) immediate-early gene promoter; polyA, the SV40 early mRNA polyadenylation signal. Bold letters were those enzymes used in plasmid construction.

The flow chart of construction of pPolyII-CAV-ΔE3-ROP18 shows that the 4.8 Kbp KpnI fragment containing the E3 region from pPolyII-CAV-2 was first cloned into pVAX I (Invitrogen), forming pVAX-E3. The ROP18 sequence (GenBank^TM^ Accession No. AM075204, 1665 bp from sequence positions 1 - 1665) was amplified by PCR from genomic DNA of *T.gondii* RH strain, with a parir of oligonucleotide primers (ROP18F, forward primer: 5' -GCTAGC ATGTTTTCGGTACAGCGGCCA-3'; ROP18R, reverse primer: 5' -AGATCTTTATTCTGTGTGGAGATGTTCCTG-3'), Nhe I and Bgl II recognition sites were introduced and underlined. The PCR product was cloned in pGEM-T easy vector (Promega, USA) and sequenced in both directions to ensure fidelity, generated pGEM-T-ROP18. The ROP18 gene was released with Nhe I and Bgl II from pGEM-T-ROP18 and cloned into peGFP-C1 (Clontech Laboratories), forming pEGFP-ROP18. The Mlu I/Ase I fragment of pEGFP-ROP18 containing the ROP18 expression cassette was filled in and cloned into pVAX-E3 digested with SspI and blunted with Klenow/dNTPs, forming pVAX-ΔE3-ROP18. The 6.3 kb fragment of Nru I and Sal I double-digested pVAX-ΔE3-ROP18, containing the ROP18 expression cassette flanked by residual E3 sequences, was cloned back into pPolyII-CAV-2 by replacing the fragment between the Nru I and Sal I enzyme sites, forming pPolyII-CAV-ΔE3-ROP18.
