# Supplementary material for: Protective efficacy of recombinant canine adenovirus type-2 expressing TgROP18 (CAV-2-ROP18) against acute and chronic Toxoplasma gondii infection in mice
Source: BMC Infect Dis. 2015 Mar 4;15:114. doi: 10.1186/s12879-015-0815-1 (PMC4397727; doi:10.1186/s12879-015-0815-1)
Supplement: Additional file 3: — The indirect immunofluorescence assay. [file 12879_2015_815_MOESM3_ESM.doc]

**Supplementary Material 3**

Briefly, MDCK cells grown on 15 mm glass coverslips in 12-well culture plates were infected with CAV-2-ROP18 or CAV-2 at an m.o.i. of 20. After 48 h infection, the coverslips were rinsed once with phosphate buffered solution (PBS, pH7.4), fixed with acetone for 10 min at room temperature and then reacted with anti-*T.gondii* polyclonal antiserum (Goat) and washed three times with PBS. The fixed monolayers were incubated at 37℃ for 30 min in a moist chamber with a FITC-labeled donkey anti-goat IgG antibody (Proteintech Group Inc., Chicago, USA). Evans blue (Fisher) was included in the secondary antibody solution as a counterstain. The coverslips were rinsed three times with PBS. Cell monolayers that bound the antibody were covered with glycerine and examined for specific fluorescence under a fluorescence microscope at 400× (Carl Zeiss, Axioplan, Germany).
