# Supplementary material for: Protective efficacy of recombinant canine adenovirus type-2 expressing TgROP18 (CAV-2-ROP18) against acute and chronic Toxoplasma gondii infection in mice
Source: BMC Infect Dis. 2015 Mar 4;15:114. doi: 10.1186/s12879-015-0815-1 (PMC4397727; doi:10.1186/s12879-015-0815-1)
Supplement: Additional file 4: — Humoral response. [file 12879_2015_815_MOESM4_ESM.doc]

**Supplementary Material 4**

In brief, microtiter plates were coated overnight at 4℃ with recombinant *T.gondii* ROP18 (200 ng/well, purifed from *Escherichia coli*) in PBS. The bound antibodies were detected by horseradish peroxidase-conjugated anti-mouse IgG, diluted in 1:2000, and IgG1, IgG2a (Serotec) 1:1500. The reaction was stopped by the addition of 1 M H_2_SO_4_, and the absorbance was measured at 490 nm using ELISA reader (Bio-TekEL × 800, USA). All samples were run in triplicate.
