# Supplementary material for: Protective efficacy of recombinant canine adenovirus type-2 expressing TgROP18 (CAV-2-ROP18) against acute and chronic Toxoplasma gondii infection in mice
Source: BMC Infect Dis. 2015 Mar 4;15:114. doi: 10.1186/s12879-015-0815-1 (PMC4397727; doi:10.1186/s12879-015-0815-1)
Supplement: Additional file 5: — Hemagglutination inhibition. [file 12879_2015_815_MOESM5_ESM.doc]

**Supplementary Material 5**

Serial 2-fold dilutions of sera were mixed with 25 μl CAV-2, titer 4 HA units, with PBS (0.01 M, pH 7.2) as control, and incubated at 37℃ for 30 min. Then 25 μl human group O red blood cells suspended in PBS was added to each well and incubated at 37℃ for 2 h. Titers were determined as the highest dilution showing at least partial agglutination.
