# Supplementary material for: Protective efficacy of recombinant canine adenovirus type-2 expressing TgROP18 (CAV-2-ROP18) against acute and chronic Toxoplasma gondii infection in mice
Source: BMC Infect Dis. 2015 Mar 4;15:114. doi: 10.1186/s12879-015-0815-1 (PMC4397727; doi:10.1186/s12879-015-0815-1)
Supplement: Additional file 6: — Lymphocyte proliferation assay. [file 12879_2015_815_MOESM6_ESM.doc]

**Supplementary Material 6**

The ROP18 (10 μg/mL) or concanavalin A (ConA; 5 μg/mL; Sigma; positive control) or medium alone (negative control) was as stimulus in the proceed of spleen cells proliferation, separately. The proliferative activity of spleen cells was measured using a 3-(4,5-dimethylthiazol-2-yl)-2,5-diphenyltetrazolium bromide (MTT, 5 mg/mL, Sigma) dye assay, according to the method described by Bounous *et al.* [16]. The stimulation index (SI) was calculated as the ratio of the average OD_570_ value of wells containing antigen-stimulated cells to the average OD_570_ value of wells containing only cells with medium. All assays were performed in triplicate.
