# Supplementary material for: Protective efficacy of recombinant canine adenovirus type-2 expressing TgROP18 (CAV-2-ROP18) against acute and chronic Toxoplasma gondii infection in mice
Source: BMC Infect Dis. 2015 Mar 4;15:114. doi: 10.1186/s12879-015-0815-1 (PMC4397727; doi:10.1186/s12879-015-0815-1)
Supplement: Additional file 7: — Cytokine assays. [file 12879_2015_815_MOESM7_ESM.doc]

**Supplementary Material 7**

To evaluate cytokine production, spleen cell-free supernatants were harvested and assayed for IL-2 and IL-4 activities at 24 h, for IL-10, IL-12 and IFN-γ activity at 72 h. The IL-2, IL-4, IL-10, and IFN-γ concentrations were evaluated using a commercial ELISA kit according to the manufacturer’s instructions (R&D Systems). Cytokine concentrations were determined by reference to standard curves constructed with known amounts of mouse recombinant IFN-γ, IL-2, IL-4, or IL-10. The sensitivity limits for the assays were 20 pg/ml for IFN-γ, 50 pg/ml for IL-2 and IL-12, and 10 pg/ml for IL-4 and IL-10.
