# Supplementary material for: Protective efficacy of recombinant canine adenovirus type-2 expressing TgROP18 (CAV-2-ROP18) against acute and chronic Toxoplasma gondii infection in mice
Source: BMC Infect Dis. 2015 Mar 4;15:114. doi: 10.1186/s12879-015-0815-1 (PMC4397727; doi:10.1186/s12879-015-0815-1)
Supplement: Additional file 8: — CTL activity. [file 12879_2015_815_MOESM8_ESM.doc]

**Supplementary Material 8**

PBMCs cultures were co-cultured with 2.5 μg ROP18 in each well of a 24-cell plate. Five days later, Sp2/0 cells (H-2^d^) transfected with CAV2-ROP18 were used as effector cells. Ten thousand target cells per well were mixed with effector cells at various effector/target (E/T) ratios in quadric-section and were incubated for 6 h. the percentage of specific lysis was calculated as: [(experimental CPM – spontaneous CPM)/(maximal CPM – spontaneous CPM)] × 100.
