# Supplementary material for: Protective efficacy of recombinant canine adenovirus type-2 expressing TgROP18 (CAV-2-ROP18) against acute and chronic Toxoplasma gondii infection in mice
Source: BMC Infect Dis. 2015 Mar 4;15:114. doi: 10.1186/s12879-015-0815-1 (PMC4397727; doi:10.1186/s12879-015-0815-1)
Supplement: Additional file 9: — Flow cytometry detection. [file 12879_2015_815_MOESM9_ESM.doc]

**Supplementary Material 9**

Primary antibody to swine leukocyte surface antigens in PBS containing 1% FCS and 0.04% sodium azide (FACS buffer) was added to wells (~1 μg/well) containing cells. Primary antibodies included phycoerythrin (PE)-conjugated anti-CD4 (PerCP-Cy5.5 conjugate clone RM4-5), and biotinylated anti-CD8γ (APC-Cy^TM^ 7 conjugate, colne 53-6.7) and anti-CD3 (8E6) incubation, cells were washed with FACS buffer, then fixed with 70% ice cold ethanol and treated with anti-CD4 and anti-CD8. The cells were incubated in a dark environment for 15 min before flow cytometric analysis.

For intracellular cytokine detection, total spleen cells (2,000,000/well) were cultured for 18 hours with 10 mg/ml ROP18 for 24 hrs. Brefeldin A (1:1000) was added to culture in the last 4 hours of incubation. Cells were washed twice in assay buffer (PBS supplemented with 1% of bovine serum albumin). Prior staining, cells were treated with 2 μg of FcγII/III receptor blocker (30 min on ice). Then, cells were submitted to surface staining with 1 mg MAb (CD4-PE, CD8-PE; eBioscience) diluted in assay buffer for 30 min at 4℃, fixed and permeabilized with Cytofix solution (BD Biosciences) for 20 min in dark. Spleen cells were stained with 1 μg of antibodies to intracellular IFN-γ and TNF-α (APC conjugate, eBioscience) for 30 min, on ice, and finally, labeled cells were washed and suspended in assay buffer. Events were acquired in a BD FACScan flow cytometer.
